# Transylvania Medical School

**Published:** 1844-07

**Authors:** 


### TRANSYLVANIA MEDICAL SCHOOL.

Our worthy contemporary, the Boston Medical and Surgical Journal, thinks it "very strange that such agreeable men," as the professors in the Lexington medical school, "after having been associated many years, could suddenly fall to loggerheads."

——"In faith, '*twas* strange, 'twas passing strange,
'Twas pitiful, 'twas wondrous pitiful."

But, unfortunately, it is but too true. These very "agreeable men" did "fall to loggerheads," and the result is that one of them, Dr. Cross, has left the school, having, it is said, been invited by his colleagues to take that decisive step. The extruded professor is understood to feel that he has been badly used by his late associates, and that he will be obliged to vindicate his character against the aspersions of certain of these "agreeable" gentlemen. Meantime, Dr. Bush sits on his seat more firmly than ever, having been advanced to the professorship of anatomy, Dr. Dudley consenting, at length, to occupy a single chair. Dr. Lawson, editor of the Western Lancet, has been elected to the professorship made vacant by the resignation of Dr. Cross. This is the *last* phasis of this changeful medical luminary. If other revolutions occur in the next month, they shall be duly noted.

